# SARS-CoV-2 variants-associated outbreaks of COVID-19 in a tertiary institution, North-Central Nigeria: Implications for epidemic control

**DOI:** 10.1371/journal.pone.0280756

**Published:** 2023-01-25

**Authors:** Oluwapelumi Olufemi Adeyemi, Nnaemeka Darlington Ndodo, Mariam Kehinde Sulaiman, Oyeronke Temidayo Ayansola, Oluwabunmi Idera Nimat Buhari, Olusola Anuoluwapo Akanbi, Oladimeji Akeem Bolarinwa, Chimaobi Chukwu, Ireoluwa Yinka Joel, Adesuyi Ayodeji Omoare, Kolawole Wasiu Wahab, Celestina Obiekea, Mikhail Olayinka Buhari, Anthony Ahumibe, Caroline Folasade Kolawole, Catherine Okoi, Olumuyiwa Babagbemi Omotesho, Nwando Mba, Oluwafemi Adeniyi, Olajumoke Babatunde, Nathaniel Akintunde, Ganiu Ayinla, Oluwatosin Wuraola Akande, Rasheed Adekeye Odunola, Mohammed Jimoh Saka, Omotosho Ibrahim Musa, Idayat Adenike Durotoye, Chikwe Ihekweazu, Ifedayo Morayo Adetifa, Abayomi Fadeyi

**Affiliations:** 1 Department of Medical Microbiology and Parasitology, Faculty of Basic Clinical Sciences, College of Health Sciences, University of Ilorin, Ilorin, Nigeria; 2 Molecular Diagnostic and Research Laboratory, College of Health Sciences, University of Ilorin, Ilorin, Nigeria; 3 National Reference Laboratory, Nigeria Centre for Disease Control, Abuja, Nigeria; 4 Department of Behavioural Sciences, Faculty of Basic Clinical Sciences, College of Health Sciences, University of Ilorin, Ilorin, Nigeria; 5 Department of Epidemiology and Community Health, Faculty of Clinical Sciences, College of Health Sciences, University of Ilorin, Ilorin, Nigeria; 6 Department of Medicine, Faculty of Clinical Sciences, College of Health Sciences, University of Ilorin, Ilorin, Nigeria; 7 Department of Pathology, Faculty of Basic Clinical Sciences, College of Health Sciences, University of Ilorin, Ilorin, Nigeria; 8 Health Services, University of Ilorin, Ilorin, Nigeria; 9 Department of Haematology, Faculty of Basic Clinical Sciences, College of Health Sciences, University of Ilorin, Ilorin, Nigeria; 10 Office of the Director General, Nigeria Centre for Disease Control, Abuja, Nigeria; Rutgers Biomedical and Health Sciences, UNITED STATES

## Abstract

The COVID-19 global pandemic is being driven by evolving SARS-CoV-2 variants with consequential implications on virus transmissibility, host immunity, and disease severity. Continuous molecular and genomic surveillance of the SARS-CoV-2 variants is therefore necessary for public health interventions toward the management of the pandemic. This study is a retrospective analysis of COVID-19 cases reported in a Nigerian tertiary institution from July to December 2021. In total, 705 suspected COVID-19 cases that comprised 547 students and 158 non-students were investigated by real time PCR (RT-PCR); of which 372 (~52.8%) tested positive for COVID-19. Using a set of selection criteria, 74 (~19.9%) COVID-19 positive samples were selected for next generation sequencing. Data showed that there were two outbreaks of COVID-19 within the university community over the study period, during which more females (56.8%) tested positive than males (47.8%) (p<0.05). Clinical data together with phylogenetic analysis suggested community transmission of SARS-CoV-2 through mostly asymptomatic and/or pre-symptomatic individuals. Confirmed COVID-19 cases were mostly mild, however, SARS-CoV-2 delta (77%) and omicron (4.1%) variants were implicated as major drivers of respective waves of infections during the study period. This study highlights the importance of integrated surveillance of communicable disease during outbreaks.

## Introduction

Coronavirus Disease 2019 (COVID-19) was first reported in Wuhan, China in December 2019 and has led to a global pandemic of colossal impact. Its aetiological agent is the Severe Acute Respiratory Syndrome Coronavirus-2 (SARS-CoV-2) [1]: an enveloped 29.7 kb positive-sense RNA virus of the family *Coronaviridae* that encodes 16 non-structural proteins at its 5’ end and the spike, nucleocapsid, membrane and envelope proteins at its 3’ end [2]. Confirmatory diagnosis of COVID-19 involves the laboratory detection of SARS-CoV-2 nucleic acid in nasopharyngeal (NP) and oropharyngeal (OP) samples of suspected cases by real-time reverse transcription-PCR (RT-PCR) [3].

RNA viruses exist as quasispecies with high rates of mutation [4] that sustain their evolution through fitness landscapes [5]. Despite available anti-COVID-19 vaccines, the COVID-19 pandemic has persisted through waves of infections driven by evolving SARS-CoV-2 variants of interest (VOI) (e.g., Eta, Iota, Kappa, Mu, Lambda, Zeta, etc) and variants of concern (VOC) (e.g., Alpha, Beta, Gamma, Delta, and Omicron), that have shaped the pandemic and response measures [6–10]. Studies have associated COVID-19 with flu-like, gastrointestinal, chemosensory, and a range of non-specific symptoms (reviewed in [11]), however, clinical distinctions have also been associated with some SARS-CoV-2 variants. A study showed that, while chemosensory symptoms were more predictive of the wild-type SARS-CoV-2 infections, persistent flu-like symptoms were more predictive of infections with the SARS-CoV-2 Alpha variant [12]. Although studies have not associated symptomatic peculiarities with the Beta and Gamma variants, the Delta variants have been associated with increased gastrointestinal symptoms [13], increased transmissibility [14], and breakthrough infections in previously vaccinated individuals [15]. Similarly, the SARS-CoV-2 Omicron variant has been associated with increased transmissibility and immune escape, thus requiring booster doses of available vaccines [16].

The COVID-19 global pandemic has provided a platform to showcase the effectiveness of an integrated approach to communicable disease surveillance [17], highlighting the benefits of strengthened indigenous components [18]. Since the beginning of the COVID-19 pandemic, Nigeria reported a first wave of COVID-19 infections in the 2020 epidemiological week (Epi week) 9, which lasted until 2020 Epi week 43; the second wave was reported from 2020 Epi week 44 to 2021 Epi week 15, the predicted third wave [19] from 2021 Epi week 26 to 44; while a fourth wave is ongoing. With increasing capacities for molecular diagnostics [20] and genomic-informed surveillance across Africa [21], epidemic control is projected to adopt enhanced dimensions. In previous reports on African responses to the COVID-19 pandemic, we highlighted the importance of timeliness, sustainability, innovativeness, and resilience using community-based approaches [18, 22], to reduce the spread of COVID-19 [22]. As a follow-up to our previous report on the roles of African institutions in the COVID-19 pandemic [22], here, we report on interventional strategies adopted by the University of Ilorin (Unilorin), Ilorin, Nigeria, that comprise the implementation of systematic public health measures which include routine molecular and genomic surveillance of SARS-CoV-2 variants associated with institutional outbreaks of COVID-19. We further discuss the public health implications of response strategies to the evolving global pandemic.

## Methodology

### Study area

Unilorin occupies a 15,000-ha landmass, located at GIS (8.4791, 4.6738) that cuts across several communities in Kwara State, North-central Nigeria. Unilorin is a community of over 56,600 students and 4,500 workers [22] that are spread across 16 faculties, a college of health sciences, and a school of preliminary studies. The health needs of the University community are managed by a healthcare centre, which has served as the hub of a university committee, which was set up in 2020 to curtail the spread of COVID-19 in Unilorin [22]. The committee implemented preventive and control activities across the University campuses, working closely with co-opted student volunteers in COVID-19 task force group. With the aid of a Tertiary Trust Fund (TETFund) grant acquired by the broad-based University committee, and in collaboration with international partners, a Molecular Diagnostic and Research Laboratory (MDRL) was set up and activated for disease surveillance in Kwara, North-Central, Nigeria.

### Ethical approval

Consent for purposeful use of patients’ anonymised data was issued by the Nigeria Centre for Disease Control (NCDC), while the study protocol and ethics were approved by the Ethical Review Committee of the University of Ilorin Teaching Hospital, Kwara, Nigeria (ERC PAN/2020/08/0041).

### Study design and study population

This study is a retrospective analysis of COVID-19 surveillance data reported at Unilorin from July to December 2021. In total, 705 nasopharyngeal/ oropharyngeal (NP/OP) samples were tested for COVID-19 by real time RT-PCR. Selected positive samples with RT-PCR cycle threshold (CT) values below 30, were further analysed by next generation sequencing to identify SARS-CoV-2 variants within the population.

### COVID-19 case-definitions

As defined by the NCDC and guided by the WHO case definitions [23], the following definitions applied to this study:

Suspected COVID-19 cases included (severely ill patients or) persons that presented with fever, cough, or dyspnoea and/ or persons, who within 14 days prior to the onset of illness had a travel history to or transit through a high-risk country with widespread community transmission of SARS-CoV-2; or persons who were in close contact with a confirmed COVID-19 case, or, had been exposed to a healthcare facility with reported COVID-19 cases.
Probable COVID-19 cases included suspected cases for whom COVID-19 tests were indeterminate, or for whom COVID-19 tests were positive on a pan-coronavirus assay; or where samples were not collected before the demise of a suspected case.
Confirmed cases of COVID-19 included symptomatic or asymptomatic person(s) with laboratory confirmation of SARS-CoV-2 infection through a nucleic acid test.

### Sample collection and handling

Teams of trained healthcare workers at the Unilorin health centre and MDRL investigated COVID-19 cases as defined by the global surveillance guidelines of the World Health Organization [24]. Respondents’ medical history and sociodemographic data were collected using the NCDC case-investigation forms, while NP and OP specimens were collected using triple packaged synthetic fibre swabs with plastic shafts. Specimens were aseptically transported to the Unilorin MDRL biorepository in viral transport media (VTM) at 2–4°C and stored at -80°C until assaying. RT-PCR positive samples with cycle threshold values (i.e., CT-values) below 30 were transported to the NCDC biorepository at 2–4°C and stored at -80°C until sequencing.

### Nucleic acid extraction and amplification

SARS-CoV-2 genomic RNA were manually extracted from NP/OP samples using the spin-column methods of extraction kits in accordance with the manufacturers’ guidelines (S1 Table). RNA extract was used immediately for RT-PCR or stored at -80°C until use. COVID-19 was diagnosed through the detection of multiple genes of SARS-CoV-2 (i.e., E-, N-, RdRp-, and ORF1-genes) of NP/OP samples collected (S1 Table) in accordance with the manufacturer’s guidelines. Prior genomic sequencing, sample integrity was revalidated by real time PCR, following re-extraction of genomic RNA. SARS-CoV-2 genomic RNA were extracted using the Life River RNA Extraction System within the automated Life River 3600 extractor (ZJ Bio-TechCo., Ltd Shanghai China) in accordance with the manufacturer’s procedure. To re-confirm sample integrity following freeze-thaw cycles, RT-PCR Ct value was re-assessed using the STANDARD M nCoV Real-Time Detection kit (SD BIOSENSOR, Inc, Republic of Korea) following the manufacturer’s instructions.

### Reverse transcription and multiplexed PCR

The RNA extracts were reverse transcribed using the LunaScript^®^ RT SuperMix Kit (New England Biolabs, Ipswich, MA, USA), followed by gene-specific multiplex PCR, using the ARTIC protocol, as described previously [25]. In summary, SARS-CoV-2 whole-genome amplification by multiplex PCR was done using ARTIC v3 primers to generate 400 base pair (bp) amplicons with 70 bp overlaps, covering the 30 kilobase SARS-CoV-2 genome.

### Library preparation and next generation sequencing

Individual libraries were generated for NP/OP samples using the NEBNext ARTIC SARS-CoV-2 FS Library Prep Kit (New England Biolabs, Ipswich, MA, USA) following the manufacturer’s instructions. Briefly, amplicons from each sample were purified and pooled as library input. This was followed by an enzymatic fragmentation through serial incubation at 37°C for 30 minutes and 65°C for 30 minutes to achieve a target size of about 250–300 bp. The ends of the fragmented amplicons were repaired, and adapters ligated, followed by kit-based purification of the fragments according to the manufacturer’s instructions. Adapter-ligated DNA fragments were amplified through 5–7 cycles of PCR using the NEBNext Multiplex Oligos (New England BioLabs, Ipswich, MA, USA) and thereafter purified according to the manufacturer’s instructions. Size distribution and concentration of the libraries were assessed on a Bioanalyzer 2100 using the High Sensitivity DNA kit (Agilent Technologies, CA, USA) and the concentration of double stranded DNA using Qubit 4 Fluorometer using Qubit dsDNA High sensitivity assay kit (Life Technologies, CA, USA). Each sample library was normalized to 4 nM concentration, and the normalized libraries were pooled and denatured with 5 μL of 0.2 N sodium hydroxide. Then, 5% PhiX control (PhiX Control v3) was spiked in a 12 pM library and loaded onto a MiSeq v3 150 cycles reagent cartridge. Sequencing was performed on the Illumina MiSeq in paired-end mode (2 × 75 bp) with v3 chemistry (Illumina, San Diego, CA, USA). FASTQ reads were generated by the Illumina pipeline using manufacturer’s guidelines.

### Sequence data analysis

The raw FASTQ data generated in Illumina MiSeq was processed and assembled using DRAGEN COVID Lineage v3.5.3 (Illumina Inc.) in Illumina BaseSpace. Phylogenetic tree was constructed using multiple genome sequence alignment MAFFT (v.7.407) [26] by mapping against the Wuhan-Hu-1 reference genome with accession: NC_045512.2. Genomic isolates of SARS-CoV-2 were assigned lineages using the PANGOLIN algorithms [27] and listed in S2 Table with their GISAID sequence IDs and dates of sampling. Subsequently, highly homoplasic sites of SARS-CoV-2 genome alignment output were masked to correct for the error prone regions of 5’-UTR and 3’-UTR without losing key sites [28]. A maximum likelihood tree was constructed using IQTREE (v.1.5.5) with 1,000 bootstrap replicates and GTR nucleotide substitution model [29–31]. The phylogenetic tree output was visualised using Figtree (v.1.4.4), annotation, and heatmap generated using a custom R script [32].

### Statistical analysis

Using the IBM SPSS Statistics 21 (Armonk, New York), binary variables were expressed as means of frequencies and percentages (%), normal continuous variables by means, while Pearson Chi-square (χ^2^) tests were carried out to measure the relationships between categorical variables. The *p*-value of <0.05 was considered statistically significant. Figures were constructed using the GraphPad Prism version 7.01 (San Diego, CA, USA). Significant differences are shown as *p*-values <0.05 (*), <0.001 (***) and <0.0001 (****).

We conducted an initial chi-square for descriptive analysis and to explore the data for further predictive model. We hypothesised that travel history, contact with COVID patients, existing medical condition and status of the university communities predict the outbreak of COVID. Any variable with significant level of p<0.25 or that were clinically important (plausibility) were included in the regression model. Multiple binary logistic regression was conducted at 95% confidence interval (95%CI). Variable were recoded to create dummy tables while interactions were checked between significant variables.

## Results

### Socio-demographic profile the COVID-19 cases

In agreement with the NCDC cases definitions for COVID-19 [23], 705 suspected COVID-19 cases of persons aged 4 to 92 years were tested for COVID-19 across the University campuses between 2021 Epi-week 30 and 52. The mean age was ~25.7 years, the median age was 21 years with a standard deviation of ±11.96 years and interquartile range of 6 years. Of the 705 suspected COVID-19 cases, 547 (~77.6%) were students, while 158 (22.4%) were non-students. In total, 372 (~52.8%) of the suspected COVID-19 cases were confirmed COVID-19 positive by RT-PCR (indeterminate tests were conclusively repeated). A significant (p<0.039) proportion of respondents tested fell within the age group of 17–35 years (85.5%), which also accounted for the highest number of COVID-19 positive cases (88.7%) recorded (Table 1). Data further showed that there were more females (54.9%) than males (45.1%) tested (p<0.05), with a higher positivity rate among the females (56.8%) compared to the males (47.8%) (p<0.05).

**Table 1:** Sociodemographic characteristics of study participants.

Sociodemographic characteristics case profiles		COVID-19 (RT-qPCR) Test Result			Χ^2^	df	*p*-value
		Positive (%)	Negative (%)	Sub-total (%)			
Type of Case	Suspected	331 (89.0)	261 (78.4)	592 (84.0)	17.817	3	0.000
	Probable	30 (8.1)	45 (13.5)	75 (10.6)			
	Confirmed	9 (2.4)	15 (4.5)	24 (3.4)			
	NR	2 (0.5)	12 (3.6)	14 (2.0)			
Age (years)	≤16	4 (1.1)	5 (1.5)	9 (1.3)	6.469	2	0.039
	17–35	330 (88.7)	273 (82.0)	603 (85.5)			
	≥36	38 (10.2)	55 (16.5)	93 (13.2)			
Gender	Male	152 (40.9)	166 (49.8)	318 (45.1)	5.739	1	0.017
	Female	220 (59.1)	167 (50.2)	387 (54.9)			
University community status	Student	304 (81.7)	243 (73.0)	547 (77.6)	7.732	1	0.005
	Non-student	68 (18.3)	90 (27.0)	158 (22.4)			
Accommodation/ location	Hostel on campus	150 (21.3)	119 (16.9)	269 (38.2)	2.251	2	0.324
	Hostel off campus	95 (13.5)	100 (14.2)	195 (27.7)			
	Others	127 (18.0)	114 (16.2)	241 (34.2)			
**Total**		**372 (52.8)**	**333 (47.2)**	**705 (100.0)**			

NR: no response. Others: non-hostel accommodation on-campus or off campus. Percentages (%) represent proportions of the total within the respective category.

As shown in Table 1, there were more students that tested positive for COVID-19 (~55.4%) than non-students (43.0%). Among the student population, 464 (~84.8%) resided within hostel facilities on campus (~49.2%) or off campus (~35.6%), while others (~15.2%) resided within non-hostel accommodations. Of the overall population, there was no significant difference between the proportion of confirmed COVID-19 cases within hostel accommodations on campus (21.3%), off campus (13.5%) or in non-hostel accommodations (18.0%) (Table 1). Similarly, non-significant proportions of individuals with confirmed COVID-19 cases reported underlying conditions or practices that could predispose for severe COVID-19 such as: pregnancy (1.1%), hypertension (2.4%), overweight (2.1%), heart disease (0.0%), asthma (3.1%), chronic lung disease (0.0%), diabetes (0.6%), smoking (0.6%), and HIV (0.6%) (Table 2).

**Table 2:** Predisposing factors to severe COVID-19.

Underlying conditions		COVID-19 (RT-qPCR) Test Result			*Χ* ^2^	df	*p-*value*
		Positive (%)	Negative (%)	Total (%)			
Pregnancy	Yes	8 (1.1)	2 (0.3)	10 (1.4)	3.249	2	0.212
	No	360 (51.1)	326 (46.2)	686 (97.3)			
	NR	4 (0.6)	5 (0.7)	9 (1.3)			
Hypertension	Yes	17 (2.4)	10 (1.4)	27 (3.8)	1.401	2	0.520
	No	351 (49.8)	318 (45.1)	669 (94.9)			
	NR	4 (0.6)	5 (0.7)	9 (1.3)			
Overweight	Yes	15 (2.1)	11 (1.6)	26 (3.7)	0.505	2	0.781
	No	353 (50.1)	317 (45.0)	670 (95.0)			
	NR	4 (0.6)	5 (0.7)	9 (1.3)			
Heart disease	Yes	0 (0.0)	0 (0.0)	0 (0.0)	0.028	1	0.742
	No	368 (52.2)	328 (46.5)	696 (98.7)			
	NR	4 (0.6)	5 (0.7)	9 (1.3)			
Asthma	Yes	22 (3.1)	20 (2.8)	42 (6.0)	0.258	2	0.905
	No	346 (49.1)	308 (43.7)	654 (92.8)			
	NR	4 (0.6)	5 (0.7)	9 (1.3)			
Chronic lung disease	Yes	0 (0.0)	1 (0.1)	1 (0.1)	1.377	2	0.620
	No	368 (52.2)	327 (46.4)	695 (98.6)			
	NR	4 (0.6)	5 (0.7)	9 (1.3)			
Diabetes	Yes	4 (0.6)	2 (0.3)	6 (0.9)	0.715	2	0.781
	No	364 (51.6)	326 (46.2)	690 (97.9)			
	NR	4 (0.6)	5 (0.7)	9 (1.3)			
Smoker	NR	4 (0.6)	5 (0.7)	9 (1.3)	1.740	2	0.444
	No	364 (51.6)	327 (46.4)	691 (98.0)			
	Yes	4 (0.6)	1 (0.1)	5 (0.7)			
HIV	NR	4 (0.6)	5 (0.7)	9 (1.3)	2.041	2	0.520
	No	366 (51.9)	328 (46.5)	694 (98.4)			
	Yes	2 (0.3)	0 (0.0)	2 (0.3)			
**Total**		**333 (47.2)**	**372 (52.8)**	**705 (100.0)**			

NR: no response. Percentages (%) represent proportions of the total within respective category.

* Fishers exact test

### Symptomatology of the COVID-19 cases

**Fig 1:**
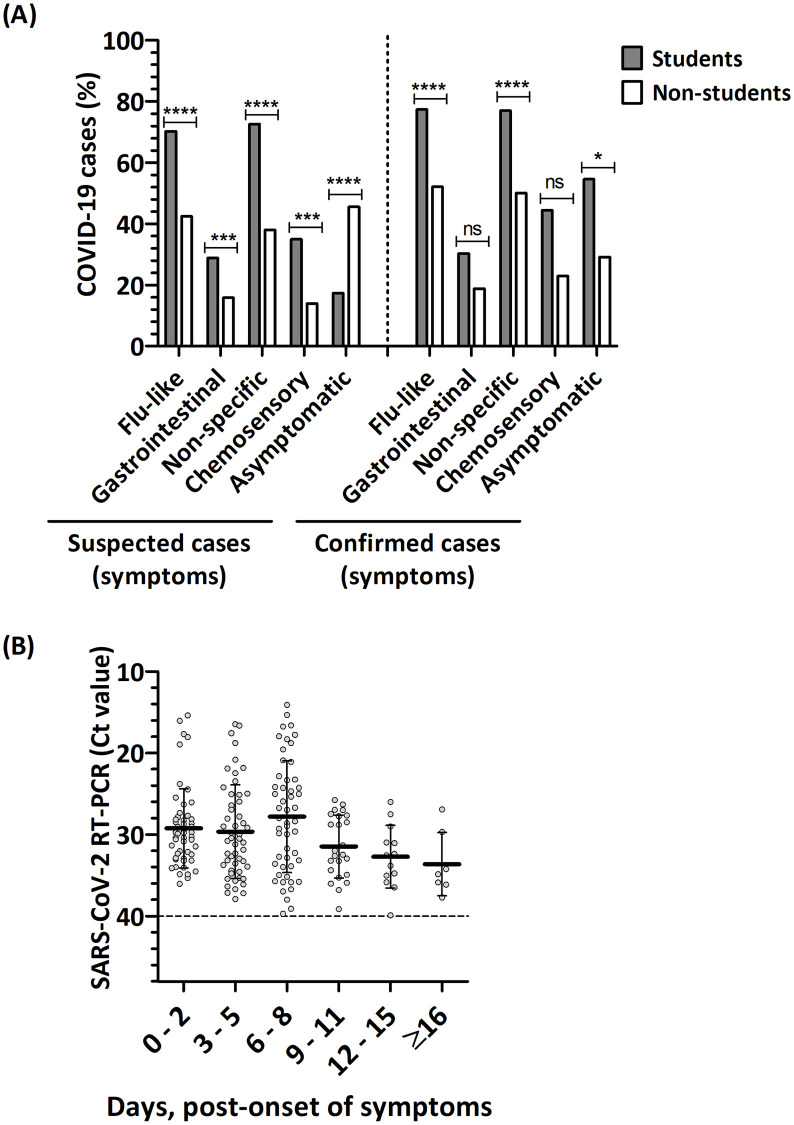
Symptomatology of COVID-19 cases. (A) COVID-19 cases are plotted on the Y-axis, while symptoms of suspected and confirmed cases are plotted on the x-axis. Bars represent percentages of students (grey) and non-students (white). Figure shows χ^2^ tests between student and non-student groups of suspected COVID-19 cases (n = 705): flu-like (χ^2^ = 85.566, df = 1, p = 0.0000), gastrointestinal (χ^2^ = 7.487, df = 1, p = 0.006), non-specific (χ^2^ = 84.532, df = 1, p = 0.000), chemosensory symptoms (χ^2^ = 8.438, df = 1, p = 0.004) and asymptomatic (χ^2^ = 12.489, df = 1, p = 0.000) (ns = not significant, ***p<0.001, ****p<0.0001). Figure also shows χ^2^ test between student and non-student groups of confirmed COVID-19 cases (n = 372): flu-like symptoms (χ^2^ = 12.906, df = 1, p = 0.0000), gastrointestinal disorders (χ^2^ = 1.368, df = 1, p = 0.242), non-specific (χ^2^ = 12.344, df = 1, p = 0.0000), chemosensory symptoms (χ^2^ = 2.647, df = 1, p = 0.104), and asymptomatic (χ^2^ = 4.399, df = 1, p = 0.036) (ns = non-significant, *p<0.05, ****p<0.0001). (B) The RT-PCR Ct values of the SARS-CoV-2 RNA are shown in reverse order on the y-axis; while the number of days, post-onset of symptoms was plotted on the x-axis against the. Scatter plot shows data points represented as grey circles; the average Ct values are shown as thick short bars; error bars represent standard deviation; dashed line represents negative Ct cut-off. Pearson’s correlation (r) of confirmed COVID-19 cases against the duration of symptoms was non-linear (r = 0.05, p = 0.471, N = 249).

In this study, COVID-19 symptoms were categorised as flu-like (i.e., one or more of fever, sore throat, runny nose, cough, and shortness of breath); gastrointestinal disorders (i.e., one or more of vomiting, nausea, and diarrhoea); non-specific (i.e., one or more of tiredness, chest pains, red eyes, headache, and any other symptom); and chemosensory (i.e., loss of smell and/or taste). Using the established COVID-19 case definitions [24], our data (Fig 1A) showed that of the suspected COVID-19 cases, flu-like (p<0.0001), gastrointestinal (p<0.01), non-specific (p<0.001), and chemosensory symptoms (p<0.01) asymptomatic (χ^2^ = 12.489, df = 1, p = 0.000) were significantly more common among the students than non-students; however, asymptomatic suspected cases were more common among the non-students than the students (p<0.001). Data further showed that flu-like (p<0.001), and non-specific symptoms (p<0.001) were significantly higher among students than non-students (p<0.05), however, students were more asymptomatic than non-students among confirmed COVID-19 cases (Fig 1). Our data further showed that about 1-in-2 students that tested positive for COVID-19 was asymptomatic (Fig 1A) (p<0.0001).

Studies have shown that peak shedding of SARS-CoV-2 particles from respiratory droplets occurs at the time of onset of symptom or ~1-week post-infection and may last ~3 weeks post-onset of symptom during which the SARS-CoV-2 genome can be detected in the upper respiratory tract by RT-qPCR [33, 34]. Therefore, we sought to investigate possible duration of viral shedding using the CT-values in correlation with the number of days since the onset of symptoms. Although not all persons that were tested for COVID-19 reported to have COVID-19-like symptoms, our data showed that, of the 343 (~43.3%) persons that reported at least one symptom, 249 were confirmed COVID-19 cases with valid Ct-values less than 40 obtained by RT-PCR (Fig 1B). On the average, the CT values of the positive RT-PCR test was ~30 within the first 5-days post-onset of symptoms; however, at 6–8 days post-onset of symptoms the CT value was ~28 and thereafter, the CT values were ~32 and ~33 at 9–11 days and 12–15 days post-onset of symptoms. Data suggests a non-linear Pearson’s correlation (r) between the number of days post-onset of symptoms and RT-PCR CT-values (r = 0.05, p = 0.471, N = 249).

### Transmission of COVID-19 within the university community

As shown in Table 3, a non-significant (p>0.05) proportion of the university community (97.9%) had no history of international travels within 14 days of reporting for their COVID-19 test. However, a significant (p<0.01) proportion of respondents (93.5%) reported no history of local travels within Nigeria in the past 14 days; of these, 338 (47.9%) tested positive, while 321 (45.5%) tested negative (p<0.01). With these findings (Table 3), we sought to investigate further, possibilities of infectious contacts of the suspected COVID-19 cases, however, our data showed that 576 (81.7%) of the suspected cases reported no history of contact with a known case in the past 14 days but at a non-significant (p>0.05) level. Data further showed (Table 3) that most of the persons that tested positive for COVID-19 were asymptomatic (p<0.0001).

**Table 3:** Contributory factors to SARS-CoV-2 transmission within the community.

Predisposing factors		COVID-19 (RT-qPCR) Test Result			Χ^2^	df	*p*-value
		Positive (%)	Negative (%)	Sub-total (%)			
International travel history within 14 days.	Yes	3 (0.4)	2 (0.3)	5 (0.7)	0.322	2	0.920*
	No	363 (51.5)	327 (46.4)	690 (97.9)			
	NR	6 (0.9)	4 (0.6)	10 (1.4)			
Local travel history within 14 days.	Yes	31 (4.4)	9 (1.3)	40 (5.7)	10.413	2	0.003*
	No	338 (47.9)	321 (45.5)	659 (93.5)			
	NR	3 (0.4)	3 (0.4)	6 (0.9)			
Contact with a known case within 14 days.	Yes	65 (9.2)	54 (7.7)	119 (16.9)	1.464	2	0.481
	No	300 (42.6)	276 (39.1)	576 (81.7)			
	NR	7 (1.0)	3 (0.4)	10 (1.4)			
Symptomatic COVID-19 case.	Yes	65 (9.2)	102 (14.5)	167 (23.7)	16.828	1	0.000
	No	307 (43.5)	231 (32.8)	538 (76.3)			
**Total**		**372 (52.8)**	**333 (47.2)**	**705 (100.0)**			

NR: no response. Percentages (%) represent proportions of the total within respective category.

* Fishers exact test

We further sought to investigate the level of association of our observations using the regression model. Briefly, a set of variables were included in the regression model with criteria stated in the methods section. Variables tested include sex, university community status, local travels, flu-like symptoms, and symptomatic individuals. As shown in Table 4, equation variables suggested that females were ~1.4 times more likely to be positive than males (p<0.05), while students were 1.6 times more likely to be positive than non-students (p<0.01). We also observed certain factors were highly predictive of COVID-19 in that, symptomatic respondents were 2.05 times more likely to test positive (p<0.001), while persons without history of local travels were 35% less likely to test positive (p<0.01) for COVID-19 than persons with history of local travels. However, of the symptoms assessed, individuals with flu-like symptoms were 1.8 times more likely to test positive for COVID-19.

**Table 4:** Predictors of COVID infection among the university community.

Variables	Adjusted Odd Ration (AOR)	95% CI	Wald statistics	p-value
Female	1.41	1.02–1.91	4.731	0.030*
University status	0.623	0.435–0.893	6.656	0.010**
Local travels	0.35	0.16–0.75	7.366	0.007**
Flu-like symptoms	1.80	1.25–2.59	9.842	0.002**
Symptomatic	2.050	1.43–2.939	15.244	0.000***

### Phylogenetics of SARS-CoV-2 genomic isolates

**Fig 2:**
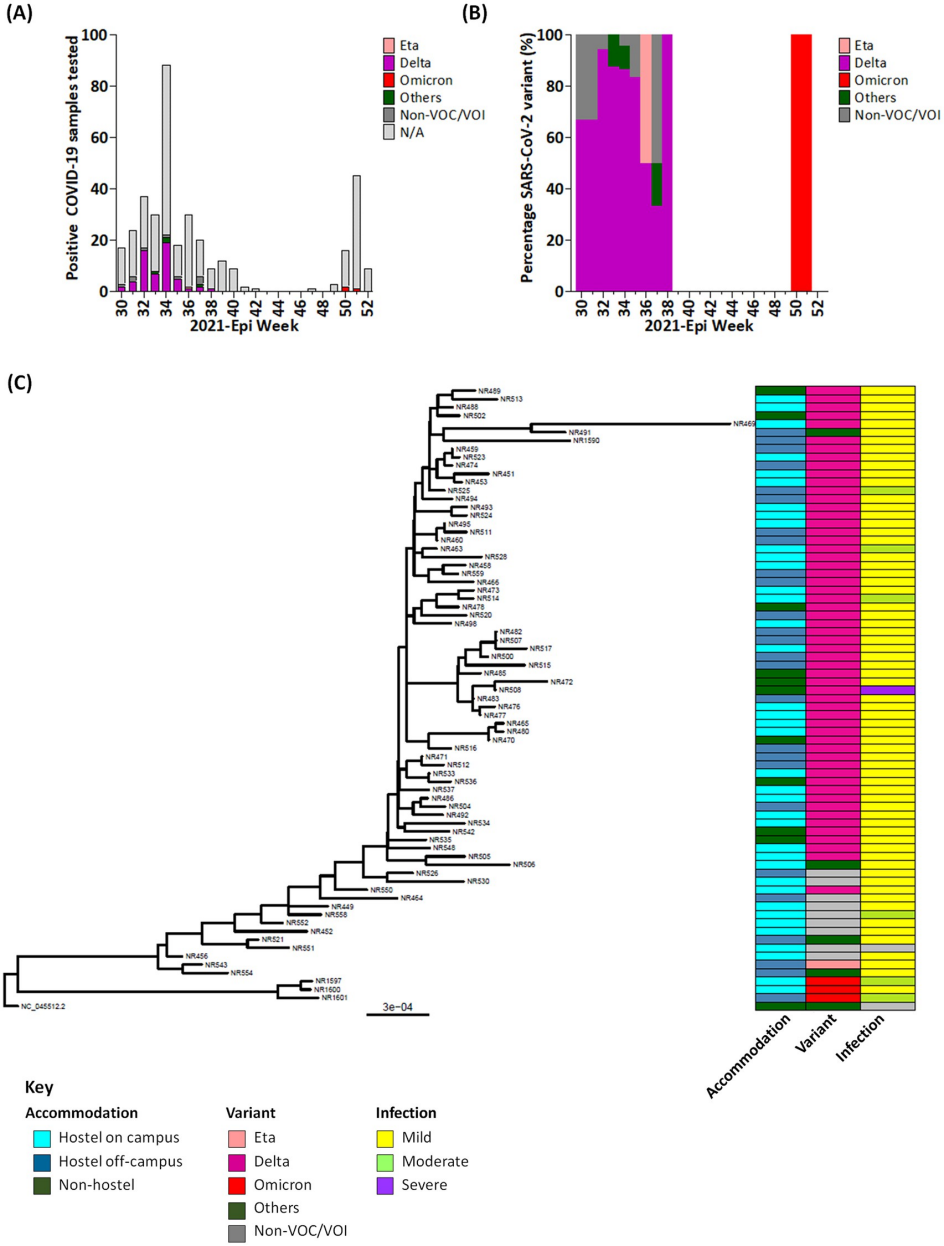
(A) Weekly positive COVID-19 samples showing distribution of variants in circulation during outbreaks. Figure shows the non-VOC/VOI (dark grey), Eta (pink), Delta (magenta) and Omicron (red) and other VOC/VOI (green). Non-sequenced samples termed not applicable (N/A) are also shown (light grey). (B) Percentage VOC/VOI Epi-weekly. non-VOC/VOI (dark grey), Eta (pink), Delta (magenta) and Omicron (red), and other VOC/VOI (green). (C) The maximum likelihood phylogenetic tree inferred from SARS-CoV-2 genomic isolates from the University community. Tips are labelled with specimen’s identification numbers corresponding with GISAID assigned accession numbers in S1 Table. The heatmap from first to third columns (left to right) indicates the accommodation type, variants of concern (VOC), and severity of infection respectively. Variant column was coloured with the major variant assigned by GISAID. The scale bar indicates distance substitution per site.

To better understand the transmission pattern of COVID-19 within the university community, we sought to investigate circulating SARS-CoV-2 variants within and around the university community through the study period as well as possible contributory roles to the reported COVID-19 outbreak within the university community. Infection severity of individuals with COVID-19 were therefore categorised into mild, moderate, and severe. Of the 372 samples that were confirmed as positive COVID-19 cases, 127 (~34.2%) samples with RT-PCR Ct values below 30 were purposively selected for next generation sequencing; however, owing to poor sample qualities, 74 samples (~19.9%) selected across the study period passed the next generation sequencing (NGS) quality assurance standards and were sequenced on the Illumina Miseq platform and constructed a phylogenetic tree to reflect the diversity and clusters among the isolates (Fig 2A and 2B). Variant’s assignment based on GASAID tool indicated the circulation of 77% delta, 4.1% omicron, 1.4% eta alongside 5.4% other lineages and sub-lineages (B, B.1, B.1.585, and B.1.1.294), which were not considered as variants of concern and 12.2% were not assigned specific lineages (Fig 2B). GISAID phylogenetic groupings of distinct variants and lineages O, G, GK, and GRA (S1 Table) were in line with the PANGOLIN tool phylogenetic groupings [27]. Of the 74 SARS-CoV-2 genomes sequenced 1 (1.4%) had severe, 6 (8.1%) mild and 65 (87.8%) had moderate and 2 (2.7%) had no symptoms. Over 90% of the delta-variant-associated COVID-19 cases were mild (Fig 2C). Furthermore, of the 74 samples sequenced, none was associated with international travels, while 9 (12.2%) samples were associated with local travels. These included 8 (88.9%) Delta variants associated with samples collected from Epi-Weeks 30–34 and 1 (11.1%) and non-VOC/VOI variant sample collected in Epi-week 32 (S1 Data). Of the SARS-CoV-2 Delta variants isolated during the period under investigation, samples associated with local travels accounted for 100%, 50% 12.5%, 14.29%, and 5.26% of the SARS-CoV-2 Delta variants isolated from Epi weeks 30 to 34, respectively, while 50% of the SARS-CoV-2 non-VOC/VOI isolated in Epi-week 32 was associated with local travels (S1 Data).

## Discussion

The adoption of a university-based triage system together with a 24-hour collaborative emergency networking at Unilorin [22] suggests a systematic and functional approach to public health surveillance within an integrated surveillance system customised to a student-majority (~77.6%) community that also had a significantly (p<0.01) higher COVID-19 positivity rate than the non-student population (Tables 1 and 4). In this study, individuals aged 18–35 years (88.7%) also accounted for the significant majority, as expected [22]. As observed in this study, most of the suspected COVID-19 cases were confirmed by RT-PCR (Table 1); thus, demonstrating a high index of suspicion as recommended [11]. Remarkably, the overall low number of confirmed COVID-19 cases with underlying conditions may have accounted for the recovery of all confirmed COVID-19 cases, which were mostly mild COVID-19 as suggested by the data provided in Fig 2C. This notwithstanding, all confirmed COVID-19 cases were managed to full recovery in accordance with the NCDC guidelines [33] and the implemented non-pharmaceutical preventive measures across the university campuses [22].

Together our findings (Table 3) suggest high possibility of asymptomatic and/ or pre-symptomatic COVID-19 spreaders (Table 3), possibly with high rates of COVID-19 transmission within the community as inferred by the steep increase in the number of cases from Epi weeks 30–34 and 49–51 (Fig 2A); however, rates of transmission were not estimated in this study. Although most confirmed COVID-19 cases reported in this study were asymptomatic, data provided (Fig 1B) suggests that shedding of SARS-CoV-2 RNA was highest at ~1 week post-onset of symptoms among symptomatic COVID-19-infected individuals, at which time they were most infectious to close contacts. This corroborates studies that suggest high levels of virus shedding ~1 week post-infection [34, 35] and further validates the importance of the highly recommended ~2-week self-isolation period for confirmed COVID-19 cases (reviewed in [36]). However, the practicality of self-isolation within hostel accommodation settings which are mostly with shared rooms and/or social spaces could be counterproductive. Furthermore, in this study, positive COVID-19 cases were more common among females than males (Tables 1 and 4). While the reason for this observation is not clear, we speculate this to be associated with social behaviours;. However, although such infection trends have been reported among the sexes in other studies, males have been shown to be more predisposed to intensive treatment unit admissions as well as COVID-19 related deaths (reviewed in [37]).

Molecular and genomic surveillance are essential tools for efficient monitoring and management of viral pandemics [38] owing to the constant evolution of viruses [4] as observed in the SARS-CoV-2 variants-driven COVID-19 pandemic [7, 8]. At early stages of the global pandemic, the evolution of SARS-CoV-2 variants is projected to have occurred through localised events within regions, such that the Alpha variant was first identified in the United Kingdom in September 2020; the Beta variant in South Africa in May 2020; the Gamma variant in Brazil in November 2020; and the Delta variant identified in India in October 2020 [7, 8]. This model limited cross-regional outbreaks of COVID-19 to imported cases [39] and was moderated by border closures and national lockdown measures that curbed the widespread of VOCs/VOIs [40, 41] within regions. As a result, the first two waves of COVID-19 in Nigeria [19] led to the closure of schools and institutions in Nigeria from March 2020 to January 2021, with potentially adverse consequences on education and learning [42] and thereby prompted a systematic reopening; thus, providing a platform for the assessment of an institutional surveillance system during the third and fourth waves of COVID-19 in Nigeria that also reflected as two outbreaks of COVID-19 within the Unilorin community (Fig 2A). However, we speculate that the concurrent identification of SARS-CoV-2 Omicron in multiple countries in November 2021 [7] following the sub-optimal vaccination rollouts across regions [43], may suggest that selection events by suboptimal anti-COVID-19 immunities [44] could have occurred in parallel across regions; however, this would require further investigations.

Bioinformatics and phylogeny tools are important in understanding microbial evolution particularly in the analysis of SARS-CoV-2 genomic evolution [45, 46]. Our data showed that the first outbreak of COVID-19 within the university community (i.e., 2021 Epi week 30 to 40) was driven by the delta variant of SARS-CoV-2, while the second wave that occurred (i.e., 2021 Epi week 49 to 52), was driven by the Omicron variant of SARS-CoV-2 (Fig 2A). In this study the phylogenetic tree was characterized by two main clusters:

One large cluster of mainly Delta variants was embedded with two sequences from other lineages (B and B.1.585) and six sequences of non-assigned lineages. We note that the appearance of B lineage clustered and shared the same node with AY.20 Delta lineage. Most of the mutations coincided with Delta variants signature mutations in spike T95I, 157del, 19R but lacked changes in spike protein G142D and P681R [47, 48]. Large cohort studies have associated increased COVID-19 severity with the SARS-CoV-2 Delta variant compared to Alpha [49] or Beta [50] variants of mostly unvaccinated individuals. In this study, most of the COVID-19 cases were reported during the first outbreak within the University community, during which more samples were sequenced to identify the variant(s) associated with the positive cases. Therefore, most cases reported in this study were associated with the SARS-CoV-2 Delta variant, which were mostly asymptomatic and/ or mild (Fig 2C). However, this could also be associated with the age range of the population (Table 1) as suggested by a scientific brief of the WHO, which associates milder cases with children, adolescents, and younger adults [51]. Nonetheless, a case of severity was observed (Fig 2C), which had sequences AY.109 Delta lineage and accounted for 13.5% of the sequences reported in this study and ~9% of the total sequences reported in Nigeria as at January 2022 (https://cov-lineages.org/lineage_list.html); therefore we cannot currently link the severity case with the lineage observed. The only two SARS-CoV-2 Delta variants isolated in Epi-week 30 were associated with local travels and this trend of SARS-CoV-2 Delta variants isolated declined subsequently until Epi-week 34 that coincided with the peak of the COVID-19 outbreak and number of SARS-CoV-2 Delta variants isolated from the university community (Fig 2A and 2B and S1 Data). This suggests possible introduction of the SARS-CoV-2 Delta variant which further circulated within the University community in the first wave of infections.

The second small cluster consists of three sequences of Omicron variant, 1 Wuhan reference strain (NC_045512.2), 1 sequence of Eta variant, 1 sequence of B.1 lineage and four sequences of non-assigned lineages. The Omicron variant phylogenetic tree was shown to produce a monophyletic clade, distantly related to other variants [52, 53]. The construction of the phylogenetic tree in this study revealed that the isolated Omicron variant was phylogenetically distant from the Delta variant while clustering with the Wuhan reference strain, Eta variant and B.1 lineage. To infer introduction and transmission of SARS-CoV-2 variants into the University community, the different accommodations occupied by participants were compared with variants observed. While different lineages and variants were found in various accommodations, no specific clustering pattern was noted indicating a community transmission of SARS-CoV-2 within the University community.

Outbreaks of COVID-19 in academic institutions Nigeria (first reported in two southwestern Universities [54, 55]) could be largely linked to the third wave of COVID-19 in Nigeria. This is due to the increasing number of cases reported soon after the reopening of the academic institutions, which led to the closure of hostel accommodations [56] and increase in the use of online and blended academic systems that combined online lectures with limited physical academic activities [57]. However, from this study, we have shown that VOC/VOI isolates from the Unilorin community were not limited to hostel accommodations on/ off campus but were spread across private accommodations of students as well as non-students (Fig 2C). This suggests high levels of community transmission of COVID-19, especially through asymptomatic and/ or pre-symptomatic individuals [58, 59]. While this is made possible through the mingling together of student and non-student populations that reside in all categories of accommodations (on- or off-campus), it further demonstrates how the university community is seen as a microcosm of the society [60]. This study further provides evidence that following the official reopening of academic institutions across Nigeria, possible outbreaks of COVID-19 within naïve communities sustained the third wave of the pandemic in Nigeria and vice versa through community transmission. While this may have a positive effect on the naivety of the population and gradually builds herd immunity through infection, it may not necessarily predispose for grave consequences if systematically approached, by targeting populations at-less-risk.

## Conclusion

Owing to the level of surveillance within the university community, this study has shown that although individuals with no history of local travels were 35% less likely to test positive for COVID-19 than those with history of local travels, further genomic surveillance suggested strong possibility of local transmission within the university community. Furthermore, this study has been able to show that positive COVID-19 cases were more associated with females (~1.4 times) than males; as well as with students (1.6 times) than non-students, and among symptomatic individuals (2.05 times) than non-symptomatic individuals. Conclusively, this study highlights significance of surveillance as a veritable infection prevention tool within an institution. It further emphasizes the importance of interventional resourcefulness and integrated surveillance using molecular and genomics systems in response to disease outbreaks as showcased by the COVID-19 global pandemic.

## Supporting information

S1 Table Commercial kits applied to nucleic acid extraction, nucleic acid and amplicon purification, reverse transcription, RT-qPCR, and NGS. (DOCX)

S2 Table Sequence ascension numbers of SARS-CoV-2 variant isolates. (DOCX)

S1 Data (XLSX)
