# Supplementary material for: SARS-CoV-2 variants-associated outbreaks of COVID-19 in a tertiary institution, North-Central Nigeria: Implications for epidemic control
Source: PLoS One. 2023 Jan 25;18(1):e0280756. doi: 10.1371/journal.pone.0280756 (PMC9876355; doi:10.1371/journal.pone.0280756)
Supplement: S1 Table — (DOCX) [file pone.0280756.s001.docx]

**S1 Table: Commercial kits applied to nucleic acid extraction, nucleic acid and amplicon purification, reverse transcription, RT-qPCR, and NGS.**

| **SN** | **Kit name (LOT number)** | **Manufacturer** | **Target(s)** |
| --- | --- | --- | --- |
|  | Quick-RNA Viral Kit | Zymo Research, USA. | RNA |
|  | DNA/RNA Purification Kit (2020015) | Daan Gene Inc. Hannover, Germany. | RNA/DNA |
|  | QIAamp Viral RNA mini-Kit (166034219) | QIAGEN GmbH, Germany. | RNA |
|  | Ribonucleic Acid (RNA) Isolation Kit (ME-0014) | Shanghai ZJ Bio-Tech Co., Ltd., Shanghai China | RNA |
|  | GeneFinder COVID-19 Plus RealAmp Kit (2010-R45-02) | OSAMG Healthcare, Korea. | SARS-CoV-2 *RdRp*- and *N*-genes, and Pan-Betacoronavirus *E*-gene |
|  | Allplex 2019-nCoV Assay (RV9120L55) | Seegene Inc., Seoul, Republic of Korea. | SARS-CoV-2 *RdRp*-gene, *N*-gene, and pan-*Sarbecovirus* *E*-gene |
|  | Novel Coronavirus (2019-nCoV) Nucleic Acid Diagnostic Kit (2020181) | Sansure Biotech Inc., Hunan, China. | SARS-CoV-2 *ORF1ab*-gene, *N*-gene |
|  | Real-time Fluorescent RT-PCR kit for detecting 2019-nCoV  (62202011143) | BGI Europe, Copenhagen, Denmark. | SARS-CoV-2 *ORF1* gene |
|  | STANDARD M nCoV Real-Time Detection kit | SD BIOSENSOR, Inc, Republic of Korea | SARS-CoV-2 ORF1ab (RdRP), and E genes |
|  | LunaScript^®^ RT SuperMix Kit (using the ARTIC v3 primers) | New England Biolabs, Ipswich, MA, USA | 30 kb SARS-CoV-2 genome |
|  | NEBNext ARTIC SARS-CoV-2 FS Library Prep Kit | New England Biolabs, Ipswich, MA, USA | SARS-CoV-2 |
|  | NEBNext Multiplex Oligos | New England Biolabs, Ipswich, MA, USA | SARS-CoV-2 |
|  | High Sensitivity DNA kit | Agilent Technologies, CA, USA | DNA |
|  | Qubit dsDNA High sensitivity assay kit | Life Technologies, CA, USA | DNA |
