# Supplementary material for: SARS-CoV-2 variants-associated outbreaks of COVID-19 in a tertiary institution, North-Central Nigeria: Implications for epidemic control
Source: PLoS One. 2023 Jan 25;18(1):e0280756. doi: 10.1371/journal.pone.0280756 (PMC9876355; doi:10.1371/journal.pone.0280756)
Supplement: S2 Table — (DOCX) [file pone.0280756.s002.docx]

**S2 Table: Sequence Ascension Numbers of SARS-CoV-2 Variant Isolates.**

| **SN** | **Sequences** | **GSAID_Epi_Isl** | **Collection date** |
| --- | --- | --- | --- |
|  | hCoV-19/Nigeria/NCDC-NR559/2021 | EPI_ISL_4743210 | 27/07/2021 |
|  | hCoV-19/Nigeria/NCDC-NR449/2021 | EPI_ISL_4743148 | 30/07/2021 |
|  | hCoV-19/Nigeria/NCDC-NR451/2021 | EPI_ISL_4743149 | 31/07/2021 |
|  | hCoV-19/Nigeria/NCDC-NR452/2021 | EPI_ISL_4743150 | 02/08/2021 |
|  | hCoV-19/Nigeria/NCDC-NR453/2021 | EPI_ISL_4743151 | 03/08/2021 |
|  | hCoV-19/Nigeria/NCDC-NR456/2021 | EPI_ISL_4743152 | 03/08/2021 |
|  | hCoV-19/Nigeria/NCDC-NR458/2021 | EPI_ISL_4743153 | 04/08/2021 |
|  | hCoV-19/Nigeria/NCDC-NR459/2021 | EPI_ISL_4743154 | 06/08/2021 |
|  | hCoV-19/Nigeria/NCDC-NR460/2021 | EPI_ISL_4743155 | 06/08/2021 |
|  | hCoV-19/Nigeria/NCDC-NR463/2021 | EPI_ISL_4743156 | 09/08/2021 |
|  | hCoV-19/Nigeria/NCDC-NR464/2021 | EPI_ISL_4743157 | 09/08/2021 |
|  | hCoV-19/Nigeria/NCDC-NR465/2021 | EPI_ISL_4743158 | 09/08/2021 |
|  | hCoV-19/Nigeria/NCDC-NR466/2021 | EPI_ISL_4743159 | 09/08/2021 |
|  | hCoV-19/Nigeria/NCDC-NR469/2021 | EPI_ISL_5440274 | 10/08/2021 |
|  | hCoV-19/Nigeria/NCDC-NR470/2021 | EPI_ISL_4743160 | 10/08/2021 |
|  | hCoV-19/Nigeria/NCDC-NR471/2021 | EPI_ISL_4743161 | 10/08/2021 |
|  | hCoV-19/Nigeria/NCDC-NR472/2021 | EPI_ISL_4743162 | 10/08/2021 |
|  | hCoV-19/Nigeria/NCDC-NR473/2021 | EPI_ISL_4743163 | 10/08/2021 |
|  | hCoV-19/Nigeria/NCDC-NR474/2021 | EPI_ISL_4743164 | 12/08/2021 |
|  | hCoV-19/Nigeria/NCDC-NR476/2021 | EPI_ISL_4743165 | 12/08/2021 |
|  | hCoV-19/Nigeria/NCDC-NR477/2021 | EPI_ISL_4743166 | 12/08/2021 |
|  | hCoV-19/Nigeria/NCDC-NR478/2021 | EPI_ISL_4743167 | 12/08/2021 |
|  | hCoV-19/Nigeria/NCDC-NR480/2021 | EPI_ISL_4743168 | 13/08/2021 |
|  | hCoV-19/Nigeria/NCDC-NR482/2021 | EPI_ISL_4743169 | 13/08/2021 |
|  | hCoV-19/Nigeria/NCDC-NR483/2021 | EPI_ISL_4743170 | 13/08/2021 |
|  | hCoV-19/Nigeria/NCDC-NR485/2021 | EPI_ISL_4743171 | 13/08/2021 |
|  | hCoV-19/Nigeria/NCDC-NR486/2021 | EPI_ISL_4743172 | 16/08/2021 |
|  | hCoV-19/Nigeria/NCDC-NR488/2021 | EPI_ISL_4743173 | 16/08/2021 |
|  | hCoV-19/Nigeria/NCDC-NR489/2021 | EPI_ISL_4743174 | 16/08/2021 |
|  | hCoV-19/Nigeria/NCDC-NR491/2021 | EPI_ISL_4743175 | 18/08/2021 |
|  | hCoV-19/Nigeria/NCDC-NR492/2021 | EPI_ISL_4743176 | 18/08/2021 |
|  | hCoV-19/Nigeria/NCDC-NR493/2021 | EPI_ISL_4743177 | 18/08/2021 |
|  | hCoV-19/Nigeria/NCDC-NR494/2021 | EPI_ISL_4743178 | 18/08/2021 |
|  | hCoV-19/Nigeria/NCDC-NR495/2021 | EPI_ISL_4743179 | 20/08/2021 |
|  | hCoV-19/Nigeria/NCDC-NR498/2021 | EPI_ISL_4743180 | 23/08/2021 |
|  | hCoV-19/Nigeria/NCDC-NR500/2021 | EPI_ISL_4743181 | 24/08/2021 |
|  | hCoV-19/Nigeria/NCDC-NR502/2021 | EPI_ISL_4743182 | 24/08/2021 |
|  | hCoV-19/Nigeria/NCDC-NR504/2021 | EPI_ISL_4743183 | 24/08/2021 |
|  | hCoV-19/Nigeria/NCDC-NR505/2021 | EPI_ISL_4743184 | 24/08/2021 |
|  | hCoV-19/Nigeria/NCDC-NR506/2021 | EPI_ISL_4743185 | 24/08/2021 |
|  | hCoV-19/Nigeria/NCDC-NR507/2021 | EPI_ISL_4743186 | 25/08/2021 |
|  | hCoV-19/Nigeria/NCDC-NR508/2021 | EPI_ISL_4743187 | 25/08/2021 |
|  | hCoV-19/Nigeria/NCDC-NR511/2021 | EPI_ISL_4743188 | 25/08/2021 |
|  | hCoV-19/Nigeria/NCDC-NR512/2021 | EPI_ISL_4743189 | 25/08/2021 |
|  | hCoV-19/Nigeria/NCDC-NR513/2021 | EPI_ISL_4743190 | 25/08/2021 |
|  | hCoV-19/Nigeria/NCDC-NR514/2021 | EPI_ISL_4743191 | 25/08/2021 |
|  | hCoV-19/Nigeria/NCDC-NR515/2021 | EPI_ISL_4743192 | 26/08/2021 |
|  | hCoV-19/Nigeria/NCDC-NR516/2021 | EPI_ISL_4743193 | 26/08/2021 |
|  | hCoV-19/Nigeria/NCDC-NR517/2021 | EPI_ISL_4743194 | 26/08/2021 |
|  | hCoV-19/Nigeria/NCDC-NR520/2021 | EPI_ISL_4743195 | 26/08/2021 |
|  | hCoV-19/Nigeria/NCDC-NR521/2021 | EPI_ISL_4743196 | 26/08/2021 |
|  | hCoV-19/Nigeria/NCDC-NR523/2021 | EPI_ISL_4743197 | 27/08/2021 |
|  | hCoV-19/Nigeria/NCDC-NR524/2021 | EPI_ISL_4743198 | 27/08/2021 |
|  | hCoV-19/Nigeria/NCDC-NR525/2021 | EPI_ISL_4743199 | 27/08/2021 |
|  | hCoV-19/Nigeria/NCDC-NR526/2021 | EPI_ISL_4743200 | 27/08/2021 |
|  | hCoV-19/Nigeria/NCDC-NR528/2021 | EPI_ISL_4743201 | 27/08/2021 |
|  | hCoV-19/Nigeria/NCDC-NR530/2021 | EPI_ISL_4743202 | 30/08/2021 |
|  | hCoV-19/Nigeria/NCDC-NR533/2021 | EPI_ISL_4743203 | 31/08/2021 |
|  | hCoV-19/Nigeria/NCDC-NR534/2021 | EPI_ISL_4743204 | 31/08/2021 |
|  | hCoV-19/Nigeria/NCDC-NR535/2021 | EPI_ISL_4743205 | 31/08/2021 |
|  | hCoV-19/Nigeria/NCDC-NR536/2021 | EPI_ISL_4743206 | 31/08/2021 |
|  | hCoV-19/Nigeria/NCDC-NR537/2021 | EPI_ISL_4743207 | 01/09/2021 |
|  | hCoV-19/Nigeria/NCDC-NR542/2021 | EPI_ISL_4578055 | 07/09/2021 |
|  | hCoV-19/Nigeria/NCDC-NR543/2021 | EPI_ISL_4578056 | 07/09/2021 |
|  | hCoV-19/Nigeria/NCDC-NR550/2021 | EPI_ISL_4578062 | 13/09/2021 |
|  | hCoV-19/Nigeria/NCDC-NR548/2021 | EPI_ISL_4743208 | 13/09/2021 |
|  | hCoV-19/Nigeria/NCDC-NR551/2021 | EPI_ISL_4743209 | 14/09/2021 |
|  | hCoV-19/Nigeria/NCDC-NR552/2021 | EPI_ISL_4578064 | 15/09/2021 |
|  | hCoV-19/Nigeria/NCDC-NR554/2021 | EPI_ISL_4578065 | 16/09/2021 |
|  | hCoV-19/Nigeria/NCDC-NR558/2021 | EPI_ISL_4630067 | 17/09/2021 |
|  | hCoV-19/Nigeria/NCDC-NR1590/2021 | EPI_ISL_8621076 | 21/09/2021 |
|  | hCoV-19/Nigeria/NCDC-NR1597/2021 | EPI_ISL_8621077 | 16/12/2021 |
|  | hCoV-19/Nigeria/NCDC-NR1600/2021 | EPI_ISL_8621078 | 17/12/2021 |
|  | hCoV-19/Nigeria/NCDC-NR1601/2021 | EPI_ISL_8621079 | 20/12/2021 |
